# Continuous developmental changes in word recognition and language learning across early childhood

**DOI:** 10.7554/eLife.109636

**Published:** 2026-07-07

**Authors:** Michael C Frank, Virginia A Marchman, Claire Augusta Bergey, Veronica Boyce, Mika Braginsky, George Kachergis, Jess Mankewitz, Stephan C Meylan, Ben Prystawski, Nilam Ram, Robert Z Sparks, Adrian Steffan, Alvin Wei Ming Tan, Martin Zettersten

**Affiliations:** 1 Department of Psychology, Stanford University Stanford United States; 2 Department of Linguistics, Stanford University Stanford United States; 3 Department of Psychology, University of Wisconsin Madison United States; 4 Department of Linguistics, University of California, Berkeley Berkeley United States; 5 Department of Communication, Stanford University Stanford United States; 6 Department of Psychology, Ludwig-Maximilians-Universität München Munich Germany; 7 Department of Cognitive Science, University of California, San Diego San Diego United States; University College London United Kingdom; Peking University China

**Keywords:** child language, word recognition, meta-analysis, longitudinal modeling, word learning, language acquisition, Human

## Abstract

Being a fluent language user involves recognizing words as they unfold in time. How does this skill develop over the course of early childhood? And how does facility in word recognition relate to the growth of vocabulary knowledge? We address these questions using data from Peekbank, an open database of experiments measuring children’s eye movements during early word recognition. In an observational study of 26 datasets from over 2500 children ages 6 months to 6 years, we show that word recognition becomes faster, more accurate, and less variable across development, consistent with a process of skill learning. Factor analysis reveals covariation of word recognition speed and accuracy with children’s vocabulary size in cross-sectional analysis. Further, across a range of longitudinal models, speed, accuracy, and vocabulary were coupled. Children with overall faster word recognition tended to show faster vocabulary growth, though developmental growth in word recognition skill was not specifically associated with growth in vocabulary. Together, these findings support the view that word recognition is a skill that develops gradually across early childhood and that this skill is deeply intertwined with early language learning.

## Introduction

Children acquiring a language are learning a body of knowledge – a set of words and the ways they are combined – but they are also learning to deploy this knowledge in the myriad complex, noisy, and fast-moving environments in which language is used. As children enter their second year, language explodes onto the scene; both vocabulary and grammatical abilities grow rapidly and in tandem (Bates et al., 1994; Frank et al., 2021). This growth in knowledge is also accompanied by changes in language processing efficiency: children become quicker and more accurate in recognizing words and matching them with their referents (Fernald et al., 1998; Peter et al., 2019; Bergelson, 2020).

Yet unlike language production, which is manifest via overt behavior, evidence for word recognition – the linking of a word form to its meaning during language comprehension – is often more subtle. Very young children may not be able to point to the correct referent of a word, but they may still have some representation of word meaning (Bergelson and Swingley, 2012). Eye tracking has thus emerged as a key method that allows the measurement of language comprehension with high temporal resolution: both adults and children reliably fixate the referent of a word soon after it is used (Fernald et al., 1998; Tanenhaus et al., 1995; Altmann and Kamide, 1999; Fernald et al., 2008; MacDonald et al., 2018). This procedure measures the general construct of word recognition by operationalizing knowledge of a word as visual attention to a specific named referent. The relative time course of fixation then can provide an index of an individual comprehender’s ability or be used to measure the difference between two stimulus conditions.

The version of this method that is used with children goes by many names, including the ‘intermodal preferential looking’ paradigm and the ‘looking-while-listening’ paradigm (LWL, the name we adopt here) (Fernald et al., 2008; Hirsh-Pasek and Golinkoff, 1996; Reznick, 1990). In LWL experiments, children are typically shown two images displayed side by side and asked to find one of them. For example, a ball and a shoe might be shown, and the child might hear ‘Look at the ball! Can you find it?’. Accuracy is then computed as the proportion of time their eyes fixate the correct image within a fixed window after the onset of the noun (‘ball’ in this case). Reaction time is (typically) computed only on trials in which the child is fixating the distractor image (the shoe) at word onset; in these cases, the average time it takes for the child to shift fixation from the distractor to the target image is used as an index of processing speed. Early work using this method showed that both children’s speed and accuracy increase rapidly across the second year (Fernald et al., 1998; Reznick, 1990). Related methods have provided a window into how children process phonological (Mani and Plunkett, 2011), morphological (Meylan et al., 2025), lexical (Swingley and Aslin, 2002), syntactic (Trueswell et al., 1999), and semantic (Mani and Plunkett, 2010; Bergelson and Aslin, 2017) information.

Familiar word recognition – as measured by LWL – is hypothesized to play a key role in language learning (Fernald et al., 2006). The idea, in a nutshell, is that the faster and more accurately a child can process incoming words, the more opportunities they have for learning. Consider a child hearing the utterance ‘Can you put the ball in the crate?’ The better the child can recognize the word ‘ball’, the better they can use this evidence to help infer the speaker’s intended meaning, allowing possible inferences about the meaning of the less familiar word, ‘crate’ (Frank et al., 2009).

Real time language processing, including word recognition, relies heavily on predictive processing, in which comprehenders integrate expectations from prior linguistic context with noisy and ephemeral incoming signals (Pickering and Gambi, 2018; Ryskin and Nieuwland, 2023). The more input a child receives, the better their predictions are likely to be, and hence the more they can learn (Fernald et al., 2006; Zettersten, 2019). Indeed, measurements of children’s language input at home are consistently associated with their vocabulary size (Hart and Risley, 1995; Anderson et al., 2021). And, in line with this predictive processing framework, one important study found that children’s word recognition speed mediated the longitudinal relationship between home language input and vocabulary growth (Weisleder and Fernald, 2013). Thus, word recognition is thought to play a key supporting role in ongoing word learning.

Familiar word recognition speed has also been used as an index of individual differences in early childhood (Peter et al., 2019; Fernald et al., 2006; Marchman and Fernald, 2008; Fernald and Marchman, 2012; Newbury et al., 2016) and beyond (Colby and McMurray, 2023; Jeppsen et al., 2025; McMurray et al., 2022). Over and above measures of vocabulary size, word recognition speed at 18 months predicts children’s language and cognitive abilities as measured by standardized tests administered at age 8 (Marchman and Fernald, 2008). Further, faster processing at 18 months is prospectively related to whether ‘late talkers’ catch up to their peers or could benefit from further intervention (Fernald and Marchman, 2012). Critically, most word recognition paradigms use words that children at the target age are reported to understand and produce. They are thus not indices of vocabulary size but rather measures of how quickly and accurately the child can recognize a familiar spoken word and use it to guide their visual attention to a referent. However, it is unknown the extent to which specific responses reflect an individual child’s general speed of language processing versus their familiarity of specific words.

Given the logistical hurdles involved in conducting eye-tracking experiments with young children, individual experiments typically recruit relatively small samples in a restricted range of ages. These samples provide neither the breadth of ages nor the number of participants needed to estimate how word recognition changes developmentally and how it connects with other aspects of early language development (see Colby and McMurray, 2023; McMurray et al., 2022 for examples of these analyses in school-aged children). To overcome these limitations, we created Peekbank, an open database of LWL data from young children, stored in a harmonized format (Zettersten et al., 2023). This dataset unifies and carefully curates a large amount of eye-tracking data from studies with infants and toddlers, representing cumulatively over 30 million individual measurements of children’s eye movements across trials and time points (dataset version: 2026.1). The Peekbank dataset allows us to gain an unprecedented view of the development of word recognition across a large sample of children.

We investigate two specific issues here. First, one influential theory posits that language learning is a process of skill learning, in which the child is learning the skill of fluent conversation with other language users (Christiansen and Chater, 2016; Chater and Christiansen, 2018). In this theory, the major information processing challenge of language learning is that incoming language is ephemeral and must be processed quickly before it is lost (the ‘now-or-never bottleneck’). On this kind of account, we should expect to see the signatures of expertise and skill learning in word recognition, which is one of the primary skills involved in processing incoming language in real time. Accuracy should change linearly with the logarithm of age, reflecting gradual asymptotic convergence to mature levels of accuracy. In addition, we might observe what is known as the ‘power law of practice’, the regularity found in many cases of skill learning that the logarithm of reaction time decreases with the logarithm of experience across participants (Snoddy, 1926; Kail, 1991; Anderson, 1982; Heathcote et al., 2000; Evans et al., 2018). Indeed, this pattern is predicted by an influential associative process model of early word learning (McMurray et al., 2012). In our case, we expect that chronological age is a proxy for experience and so the logarithm of reaction time should decrease linearly with the logarithm of age. Finally, trial-to-trial variability in both speed and accuracy should decrease with increasing expertise, as is found in studies of motor expertise (Todorov and Jordan, 2002).

Second, previous findings have provided limited and sometimes conflicting evidence on the concurrent and predictive relations between word recognition and language learning. Initial reports showed strong prospective relationships between both speed and accuracy and later vocabulary growth (Fernald et al., 2006), with replications in infants born preterm (Marchman et al., 2016) and late talkers (Fernald and Marchman, 2012). Subsequent studies have primarily focused on speed of processing and found more mixed results, with reaction time measures found to be only inconsistently related to later vocabulary outcomes (Peter et al., 2019; Newbury et al., 2016; Lany, 2018). A larger dataset should allow us to make a more definitive test of the presence of these relationships. Further, by examining the relationship between speed, accuracy, and vocabulary, it should be possible to assess the extent to which processing speed specifically plays a role in vocabulary growth.

Across both of these issues, the contribution of our work here lies in the detailed quantitative description of development. Nearly every theory of language learning assumes *some* role for continuous developmental change in word recognition, but these assumptions have not previously been anchored to specific measurements. Hence neither the functional form of the assumed changes nor their concurrent and predictive relationships to vocabulary have been quantified. We leverage the Peekbank dataset to accomplish these goals.

## Results

We retrieved data from Peekbank, focusing on data from monolingual English-speaking children ages 6 months to 6 years and on simple word recognition trials in which children were shown two pictures of concrete objects and heard a label for an object (typically embedded in a simple carrier phrase such as ‘Look at the …’). While other experimental manipulations and languages are included in the database, we narrowed our sample to English-speaking children because they are well-represented across our age range and excluded manipulations which aimed to capture phenomena other than simple concrete noun reference (e.g., adjective comprehension or novel word learning). These criteria yielded 26 datasets, including 2555 children and 4124 administrations of the LWL procedure (some datasets were longitudinal or involved multiple closely spaced testing sessions).

Table 1 shows the characteristics of individual datasets (see also Appendix 1, Dataset description). The size of the combined dataset, the unified data processing pipeline, and the fact that individual studies used very similar implementations of the LWL experimental paradigm all allowed us to make a more detailed study of the development of word recognition than has previously been possible. While our analyses are exploratory in nature, they are guided by the two hypotheses outlined above: the presence of (1) signatures of skill learning in word recognition, and (2) linkages between word recognition and vocabulary.

**Table 1.** Characteristics of included datasets from Peekbank. ‘Admins’ denotes separate experimental sessions. ‘CDIs’ refers to whether the dataset contains parent report vocabulary data from the MacArthur–Bates Communicative Development Inventory.

	Dataset name	Pct trials (%)	*N* subjects	*N* admins	Mean age	Min age	Max age	Avg trials	Avg RT trials	CDIs	Longitudinal
1	Adams et al., 2018	24.1	69	711	23.58	13.00	38.00	18.65	7.92	x	x
2	Fernald and Marchman, 2012	20.1	122	679	23.91	17.00	32.00	16.23	6.91	x	x
3	Weaver et al., 2024	8.0	141	247	15.74	13.50	23.60	18.21	6.78		x
4	Fernald et al., 2013	7.4	80	178	20.04	17.00	26.00	23.17	9.16	x	x
5	Fernald et al., 2006	6.4	63	229	19.68	15.00	25.00	15.28	6.06	x	x
6	Bergelson and Swingley, 2012	2.9	84	84	11.76	5.98	20.83	18.96	6.74	x	
7	Yurovsky et al., 2013	2.8	385	385	33.77	12.20	59.51	5.89	2.63		
8	Borovsky and Peters, 2019	2.8	79	79	18.27	17.00	20.00	19.24	0.00	x	
9	Potter and Lew-Williams, 2024	2.7	67	67	23.76	21.00	27.00	21.69	7.92		
10	Yurovsky et al., 2017a	2.6	315	315	36.40	8.40	60.00	6.27	2.87		
11	Yurovsky and Frank, 2017b	2.6	282	282	25.64	12.59	58.65	5.91	2.79		
12	Weaver and Saffran, 2026	2.4	64	64	18.82	18.10	20.10	21.82	8.19	x	
13	Yoon et al., 2015	2.0	194	194	42.30	13.20	60.00	6.72	2.98		
14	Mahr et al., 2015	2.0	29	29	20.83	18.10	23.80	37.00	13.62	x	
15	Garrison et al., 2020	1.7	35	35	14.46	12.00	18.00	27.76	9.41	x	
16	Ronfard et al., 2022	1.3	40	40	19.95	18.00	24.00	18.56	7.62	x	
17	Bacon and Saffran, 2022	1.3	38	38	22.87	22.00	24.00	18.08	8.00	x	
18	Perry and Saffran, 2017	1.2	42	42	20.45	19.00	22.00	15.45	5.43	x	
19	Pomper, R. & Saffran, J. R. (2017). Do infants learn to associate diminutive forms with animates? [unpublished raw data]. University of Wisconsin-Madison.	1.1	76	76	16.70	14.00	19.00	8.71	3.38		
20	Swingley and Aslin, 2002	1.1	50	50	15.09	14.13	16.00	11.70	3.79	x	
21	Frank et al., 2016	0.8	105	105	33.89	12.13	59.84	6.15	2.69		
22	Pomper and Saffran, 2016	0.8	60	60	44.27	41.00	47.00	7.62	3.30		
23	Moore and Bergelson, 2022	0.7	29	29	18.11	16.12	20.03	12.97	4.89	x	
24	Pomper, R. & Saffran, JR. (2015). Modulating attention to different features of objects during word learning [Unpublished Raw Data]*.* University of Wisconsin-Madison.	0.6	25	25	40.04	38.10	42.00	13.96	5.17		
25	Pomper and Saffran, 2019	0.4	44	44	40.11	38.00	43.00	5.32	2.34		
26	Pomper and Saffran, 2018	0.4	37	37	39.46	37.80	43.00	5.47	2.79		
	Total	100	2555	4124	25.38	5.98	60.00	14.88	5.51	14	5

### Speed and accuracy of word recognition increase

**Figure 1.**
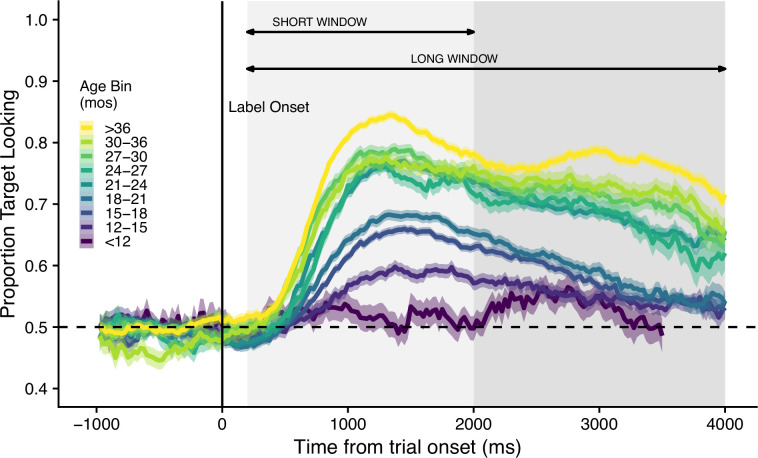
Time course of word recognition at different ages. The *x*-axis shows time (in ms) from the onset of the target label (vertical solid line). Colored lines show the average proportion target looking as a function of time from pre- and post-label onset at each age bin (in months). Age bins are larger for older children due to decreased data density. The dashed horizontal line represents chance looking. Error bands represent standard errors of the mean. Gray backgrounds highlight the short and long time windows used in subsequent analyses. The data within the figure is filtered such that (**a**) participants are required to contribute at least 5 observations and (**b**) there must be at least 50 participants contributing to each time bin within an age group.

We began by examining developmental changes in children’s word recognition. Figure 1 depicts the average time course of target looking at different ages across all datasets (not controlling for any variation in items and procedures across age groups). Intuitively, these time courses show gradual increases in accuracy (more target looking; computed as the ratio of target to target plus distractor looking) and speed (faster looking to the target after hearing a label) as age increases. To characterize age gradients in speed and accuracy across children, we computed both RTs (reaction times) and accuracies (proportion looking at the target image) following standard practices in the literature (Fernald et al., 2008). Reaction times were computed only on trials for which the child was fixating the distractor at the point of disambiguation (label onset), and were defined as the time from label onset to the first fixation on the target image (see Appendix 2, Reaction times, including further details on how reaction times were computed in Appendix 2.1 and discussion of issues surrounding distinguishing ‘correct’ versus ‘incorrect’ trials when computing looking-based reaction times in Appendix 2.2).

Because there is no consensus about the length of time windows for the computation of accuracy, we considered both a shorter window (from 200 to 2000 ms after noun onset) and a longer window (from 200 to 4000 ms). For each window, we averaged all fixations within the window to compute a continuous proportion of target looking between 0 (no fixation on the target during the window) and 1 (total fixation on the target during the window) on every trial. In this initial analysis, we treat observations of RT and target looking as direct measures of the constructs speed and accuracy (see Appendix 4, Test–retest reliability); in subsequent analyses we estimate latent variables representing these constructs.

Our first question was about the functional form of the relationships between age, speed, and accuracy (see Appendix 5, Pairwise correlations of main measures, for raw pairwise correlations between variables). We began by fitting linear mixed-effects models predicting speed and accuracy on each trial across the full dataset with random slopes of child age nested within study (modeling item and procedural variation across studies) and random intercepts by participant (see Appendix 8, Mixed-effects model specifications, for further details on these specifications). We compared models that included both long and short accuracy windows, as well as logarithmic and linear effects of age, and logarithmic and linear transformations of RT (see Appendix 3, Checks on data distributional assumptions, for further analyses and discussion of these modeling choices). The best fitting model of accuracy predicted long window accuracy as a function of the logarithm of age; the best fitting model of speed predicted log RT as a function of log age as well (see Appendix 6, Functional form model comparison, and Appendix 7, Power law fits). Because long window accuracies were more correlated with other variables and showed clearer age gradients, we focus on these in our analyses.

**Figure 2.**
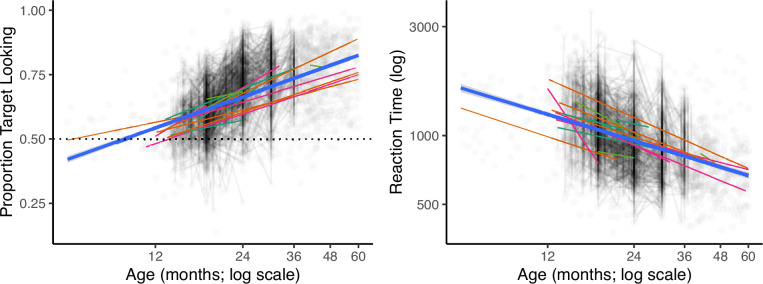
Participant-level target looking and reaction time (log), plotted by age (log). Longitudinal datapoints are connected by lines. The solid blue line shows a linear fit and associated confidence interval. Thin colored lines show linear fits for those datasets spanning six or more months of age. The dashed line for accuracy shows chance-level looking (0.5).

Figure 2 shows these age gradients. Log RT decreased significantly with age, reflecting increasing speed ($\hat{\beta}=-0.13$, 95% CI [−0.16, −0.11], $t(18.93)=-12.23$, *p* < 0.001) and accuracy also increased significantly with age ($\hat{\beta}=0.07$, 95% CI [0.06, 0.08], $t(20.17)=13.05$, *p* < 0.001). In sum, we see continuing improvements in word recognition across the full age range in our dataset that appear roughly linear in the logarithm of age. These logarithmic relationships follow theoretical expectations that both speed and accuracy should gradually asymptote to mature levels of performance, as seen in skill learning more generally (Snoddy, 1926; Anderson, 1982).

### Variability of word recognition decreases

**Figure 3.**
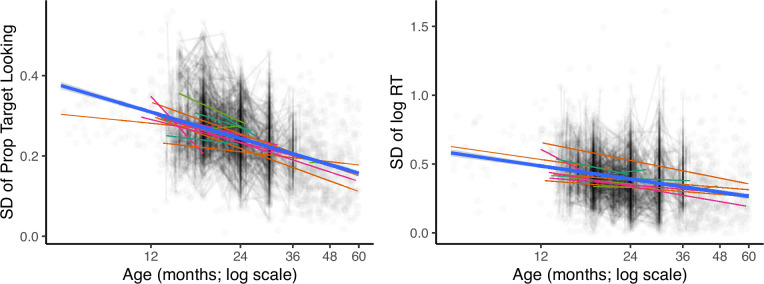
Participant-level variability in target looking and reaction time (log RT), plotted by age (log). Plotting conventions are as in Figure 1.

One further hallmark of increasing skill is a decrease in task-relevant variability (Todorov and Jordan, 2002). Both within and across datasets, within-individual variation in speed and accuracy decreased across the developmental range we examined (Figure 3). We fit mixed-effects models predicting the standard deviation of both speed and accuracy for each testing session for each participant, including random slopes of log age nested within dataset and random intercepts for each participant. For both speed and accuracy, within-individual variability decreased with age (speed: $\hat{\beta}=-0.05$, 95% CI [−0.06, −0.03], $t(16.33)=-7.19$, *p* < 0.001; accuracy: $\hat{\beta}=-0.04$, 95% CI [−0.04, –0.03], $t(12.29)=-10.45$, *p* < 0.001). Thus, as well as being faster and more accurate, older children were more consistent in their real-time word recognition than younger children.

### Speed and accuracy relate to vocabulary size

We were next interested in whether the various aspects of word recognition – including speed, accuracy, and the variability of each of these – were related to other aspects of early language ability. In our prior analyses, chronological age acts as a proxy for greater language experience and larger vocabulary as well as a host of other correlated developmental changes in cognition. Now we explicitly explore relations to vocabulary growth and the triadic relationship between age, word recognition, and vocabulary.

Of the studies in our database, 14 gathered parent reports about children’s early vocabulary using the MacArthur–Bates Communicative Development Inventory (CDI), a popular survey instrument that provides a reliable and valid estimate of children’s early vocabulary (Frank et al., 2021; Marchman et al., 2023). Different forms of the CDI can be used to measure either receptive and expressive vocabulary (for children up to 18 months) or expressive vocabulary only (for children 16–30 months).

We fit a series of factor analytic models to explore the dimensionality of the parent report and child LWL data. Our goal in these analyses was to understand the underlying relatedness of the various measures of word recognition and vocabulary, and in particular to assess the evidence for (1) whether the speed, accuracy, and variability measures described above all index the same underlying language processing construct and (2) the nature of the relation between this construct (or set of constructs) and early vocabulary. We initially add age as an additional variable to our models to explore whether this factor structure relates to age; later we treat age as a predictor of latent factors. We begin developing models using all data, treating each observation as independent even if it comes from a longitudinal study; this assumption is equivalent to asserting an invariant factor structure across development (for a test of this assumption, see Appendix 10, Factor analysis on first administrations). In subsequent models, we relax this assumption and explore longitudinal growth.

Initial exploratory factor analysis using parallel analysis to select the number of factors suggested that three factors explained substantial variance in the data (see Appendix 9, Factor analysis). To better accommodate missing data under the assumption of data missing at random (e.g., missingness due to the age sampling schemes of the various datasets), we used confirmatory factor analysis with full information maximum likelihood to find the best set of loadings. The best fitting model was a three-factor model with factors for speed (RT and RT variability), accuracy (proportion looking to target on each trial and associated variability of this measure), and vocabulary (comprehension and production from the CDI). Fit statistics for this model were generally good (Comparative fit index: 0.98, RMSEA: 0.06); see Appendix 11, Alternative factor structures.

**Figure 4.**
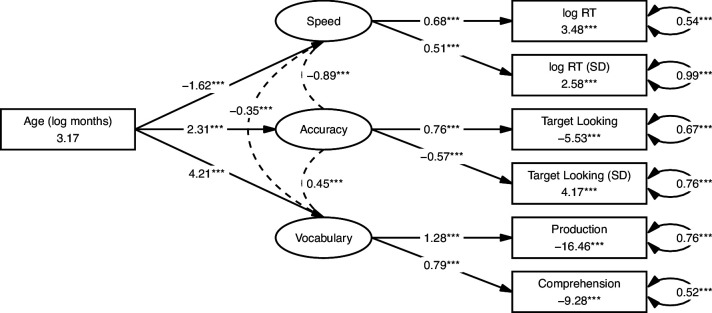
Structural equation model showing the three-factor factor analysis with a regression of each latent variable on the logarithm of age. Observed variables are notated as squares and latent variables are notated as circles. Factor loadings and regression coefficients are shown with straight, solid lines; covariances are shown with dashed lines; residual variances are shown as solid circular connections. Stars show conventional levels of statistical significance, e.g., * indicates p < 0.05, ** indicates p < 0.01, and *** indicates p < 0.001. Covariances reflect age-residualized correlations between variables.

Figure 4 shows a regression model fit to this confirmatory factor analysis, with log age predicting each latent variable. This regression model allows interpretation of the covariances between latent factors as partial correlations (controlling for age). The non-age related variance of all three latent factors was significantly related to that of the other factors. Speed and accuracy showed strong negative covariance (*β* = –0.89, *SE* = 0.03, *p* < 0.001), as expected since they are derived from the same data. Importantly, there was also weaker but significant covariation between RT and vocabulary (*β* = –0.35, *SE* = 0.04, *p* < 0.001) and accuracy and vocabulary (*β* = 0.45, *SE* = 0.03, *p* < 0.001). This model supports the idea that variation in speed and accuracy of word recognition is related to individual differences in parent-reported vocabulary beyond the effects of age. Further, the broader set of analyses supports a factor structure in which speed and accuracy (and their associated variabilities) are related but distinct aspects of word recognition, rather than being measures of one single construct. These analyses treat all data as between person, however, rather than modeling change in these factors within individuals.

### Speed of processing relates to vocabulary growth

We next investigated within-person relationships between LWL and vocabulary. In particular, we investigate two different (but not mutually exclusive) hypotheses about how word recognition skill could support word learning. First, early word recognition skill could lay a foundation for later vocabulary growth – we test this question first using a series of longitudinal growth models testing whether individual variability in processing speed predicts later increases in productive vocabulary. A second, stronger version of this hypothesis is what we call a ‘virtuous cycle’ model of the relationship between processing speed and vocabulary growth, in which not only baseline word recognition skill, but also children’s improvements in this skill are related to faster growth in vocabulary; we test this hypothesis using longitudinal structural equation models.

To investigate the first hypothesis, we began by fitting longitudinal growth models to the full dataset (though note that the same conclusions hold when restricting the data to only those children with multiple LWL sessions). We first reproduced the analysis reported in Fernald and Marchman, 2012, in which between-person differences in longitudinal growth in productive vocabulary were predicted based on between-person differences in speed during the initial session of the study. We fit a mixed-effects model predicting growth in vocabulary as a quadratic function of age, RT at study initiation ($t_{0}$), and their interaction (as well as random effects of age nested within participant and also age nested within dataset). This model revealed a significant effect of $t_{0}$ RT ($\hat{\beta}=-0.14$, 95% CI [−0.19, −0.08], $t(530.16)=-4.85$, *p* < 0.001) and an interaction between $t_{0}$ RT and the quadratic age predictor ($\hat{\beta}=2.00$, 95% CI [1.04, 2.96], $t(545.67)=4.07$, *p* < 0.001). This analysis suggests that children with faster initial RTs show both larger vocabularies and faster vocabulary growth over time.

**Figure 5.**
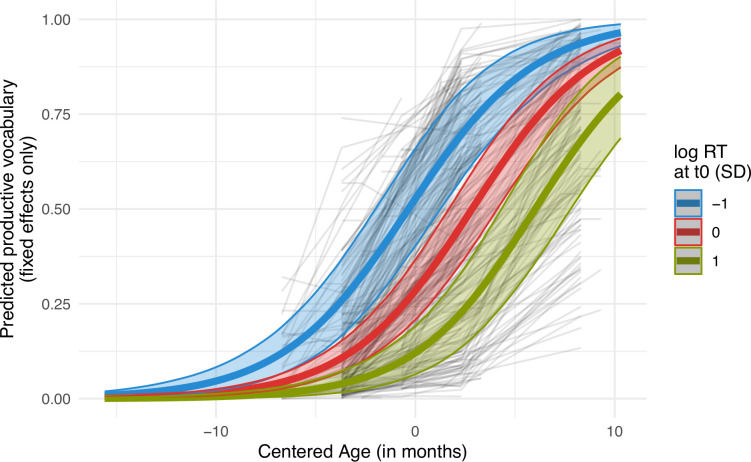
Growth curves from a logistic growth model showing predicted productive vocabulary growth for children with initial reaction times one SD faster than the mean (blue), at the mean (red), and one SD slower than the mean (green). Individual longitudinal trajectories are shown in light gray. Solid lines show global model estimates, and colored regions indicate 95% credible intervals.

We confirmed this analysis using a non-linear growth model with a logistic shape, which provides a better fit to vocabulary size within a fixed-length form than the quadratic model (see Appendix 12, Non-linear growth model) (Frank et al., 2021). Figure 5 shows predictions from this model, confirming the differentiation of growth curves for children with higher and lower initial reaction time.

**Figure 6.**
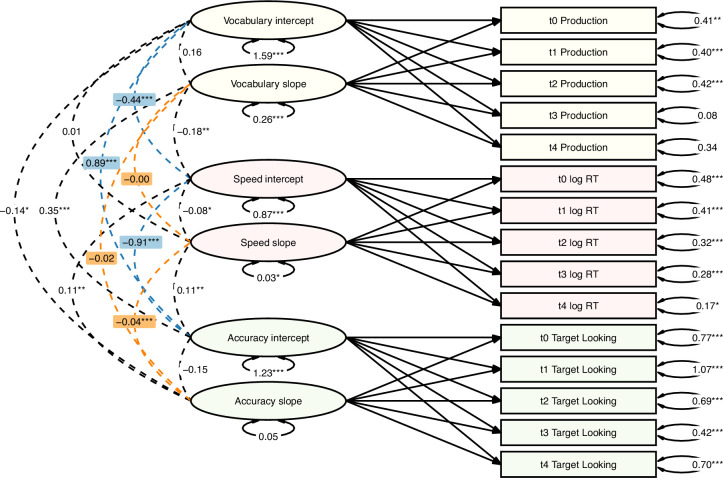
Structural equation model showing longitudinal couplings between growth parameters.

On the other hand, it is possible that differences in predicted growth trajectories are due to coupling between vocabulary size and language processing across the entire developmental period, rather than a predictive relationship specifically between $t_{0}$ RT and vocabulary growth (i.e., the ‘virtuous cycle’ model). To test this relationship, we used longitudinal structural equation models. We separated the longitudinal speed, accuracy, and vocabulary data into 2-month bins spanning up to 10 months from the initial measurement (i.e., $t_{0},\dots,t_{4}$) and fit individual growth across each of these variables. We used full information maximum likelihood to handle the substantial missing data caused by the different longitudinal sampling schemes of studies in our dataset (see Appendix 13, SEM longitudinal missingness). The fitted longitudinal model is shown in Figure 6. Overall fit statistics were generally acceptable (Comparative fit index: 0.89, RMSEA: 0.03, RMSEA *p*-value: >0.999).

Our key question of interest concerned coupling among the (latent) intercepts and slopes of these growth models. Consistent with our earlier analysis showing that faster processing is related to vocabulary growth, we saw significant between-person coupling between processing speed intercepts and vocabulary growth slopes (*β* = –0.18, *SE* = 0.06, *p* = 0.001) as well as a variety of other between-person couplings. On the other hand, there was not significant coupling between *growth* in speed and *growth* in vocabulary (*β* = 0.00, *SE* = 0.02, *p* = 0.872). This null effect could be interpreted as being consistent with these abilities growing independently, but there are other possibilities. First, the longitudinal data we had might not have allowed sufficiently precise estimates of growth slopes, or second, since vocabulary growth is non-linear, the linear model we used here might not have captured coupling among non-linear aspects of developmental change.

In sum, these findings provide evidence consistent with the claim that differences in processing speed are related to differences in the rate of age-related change in vocabulary (Fernald et al., 2006; Weisleder and Fernald, 2013). Children with greater skill in word recognition learn words faster. However, we did not find evidence for the stronger version of this claim: in neither the non-linear growth model nor the linear SEM did we find evidence that increases in speed were related to increases in vocabulary size. Thus, our findings do not support a ‘virtuous cycle’ model in which increases in recognition specifically lead to increases in vocabulary size.

## Discussion

How does word recognition change across early childhood and how does it relate to language learning? We investigated these questions using a new, large-scale dataset of developmental eye-tracking measurements compiled across many prior studies. The age gradients for speed and accuracy indicated that both improve asymptotically. Gradients for recognition speed were consistent with the log–log relationship associated with the ‘power law of practice’, that is, with a gradual convergence to mature levels of processing efficiency. Further, the age gradient suggested that trial-to-trial variability decreases with age, consistent with both the literature on skill learning (Todorov and Jordan, 2002) and other work on developmental changes in variability (Judd et al., 2021; Galeano Weber et al., 2018; Kautto et al., 2024). Speed and accuracy were both related to vocabulary size concurrently, and processing speed was also related longitudinally to later vocabulary growth.

Together, our findings are consistent with theories that posit that language learning is a process of skill acquisition, in which children become adept at quickly converting ephemeral signals into meaning (Christiansen and Chater, 2016). This skill develops gradually over the course of early childhood and supports word learning. Further, our results point to consistency between skill development in early childhood and the continued refinement of language processing and language knowledge during middle childhood (Colby and McMurray, 2023; McMurray et al., 2022).

By aggregating data from many pre-existing studies, we were able to overcome the limitations of prior investigations, which typically had sample sizes at least an order of magnitude smaller than ours. Our approach was to build on the time-consuming and meticulous data collection from previous infant and toddler eye-tracking studies – representing cumulatively many thousands of hours of in-lab data collection and hand-annotation of the resulting videos of child looking behavior – by harmonizing these data into a single, large-scale database. This approach illustrates how building harmonized databases can be especially powerful when composed of high-effort and high-quality datasets that are smaller in scope, maximizing the impact of previous data collection efforts and allowing us to ask broader questions about developmental change (Frank et al., 2021). In contrast to individual studies, which typically have at best the statistical power to test one or two specific contrasts, our ‘big data’ approach provided the sample sizes necessary to explore the relationships between different variables. Because early language is so variable, these kinds of samples – with thousands, rather than dozens of children – are likely to be required to gain further insight into the psychometrics of early language learning (Frank et al., 2021; Bergelson et al., 2023; Frank et al., 2020).

Our approach is both observational and exploratory. Thus, we cannot untangle the range of different causal models that explain the variation we observed. First, early word recognition skill could lead to faster word learning, but faster children could also be faster due to their larger vocabulary and stronger lexical representations. These two causal directions could also interact reciprocally, leading to a ‘rich get richer’ process in which children with larger vocabularies process faster, and their faster processing helps them increase their vocabulary size more rapidly. Finally, a third shared factor – perhaps general cognitive ability – could underpin both processes. Our cross-sectional data cannot distinguish these hypotheses even in principle (VanderWeele and Batty, 2023), and our longitudinal data are likely too sparse to distinguish such complex causal models. Future work must also explore how the functional forms we observed here between individuals reflect processes of within-person change. Although the Peekbank dataset includes a variety of longitudinal data, most reflect a small number of measurements; denser longitudinal data collection is required to better estimate within-person growth models.

The relationships we report are derived from models that account for variation across datasets, suggesting that our qualitative conclusions are robust to cross-laboratory variation. Nevertheless, these findings are still limited in their generalizability by the convenience samples that were used in most of the studies aggregated in Peekbank. These studies typically (but not always) represent children from well-educated parents living in university-adjacent communities. We would not expect that specific numerical parameters estimated in our aggregate convenience sample would generalize to other samples.

More broadly, our results here suggest the continued importance of the LWL paradigm as an index of children’s language processing abilities. If language learning is, at least in part, a process of skill learning, then measurement of this skill in larger samples provides a critical window into understanding the remarkable process of language learning.

## Materials and methods

### Data

We included information from 2555 unique participants across 26 datasets. Dataset information is given in Table 1. Although experiments in Peekbank include a variety of different experimental manipulations, we analyzed only data from standard, simple word recognition trials; these trials were sometimes the main focus of the original studies and sometimes constituted control conditions for experiments with more complex manipulations. Requirements for being considered a standard word recognition trial included that (1) the target word was familiar (also no part-words); (2) the target word was the first point of disambiguation and appeared only once; (3) the target word was embedded in a well-formed, grammatical carrier phrase; (4) there was no informative language presented prenominally (e.g., semantically informative verbs, adjectives); (5) there were no nonsense words presented anywhere during the trial (including the carrier phrase); (6) there was no language-, speaker-, or accent-switching within trial; (7) the auditory stimulus included no intentional background noise or audio filtering; (8) both target and distractor items were familiar objects; (9) no novel visual stimuli (i.e., experimenter-created artificial items or items selected to be entirely unfamiliar) were visible; and (10) the target referent was the focal object in the target image and there were no additional focal objects competing for attention within the target image (e.g., if the target word was ‘orange’ and the image depicted an orange on a plate, this was considered a standard trial; if however the image depicted both an apple and an orange on a plate, this was not considered standard). We focus here on English purely for practical reasons – the Peekbank dataset at present contains limited data from other languages.

We excluded trials entirely if they were missing data on more than 50% of time points, and excluded RTs if they were based on fewer than 50% of time points in the short analytic window (200–2000 ms). We also removed RTs shorter than 367 ms, as these were unlikely to be generated based on the specific linguistic stimulus. We then excluded participants from the analysis if they contributed fewer than four accuracy measurements or fewer than two reaction time measurements. At the participant level, these steps together led to 21.40% missingness for RTs and 8.80% missingness for long window accuracies.

### Analytic methods

We used lme4 to fit linear mixed-effects models, brms to fit non-linear growth models, and lavaan to fit structural equation models. Random effects structures for each model are given in text; full model specifications are available in the appendices (Appendices 8, 9, and 12) and in the reproducible code for this paper, available in the linked repository. To aid interpretability, all variables were standardized (*z*-scored) prior to inclusion in structural equation models.

## Figures and Tables

**Appendix 1—figure 1.**
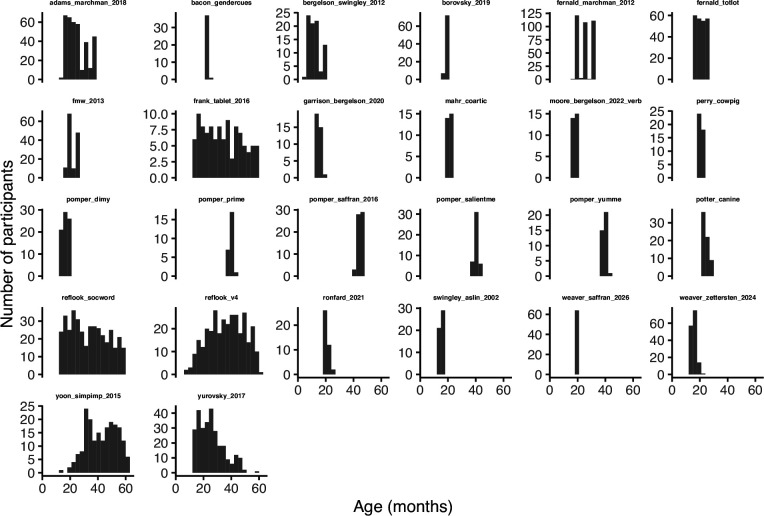
Age distribution of unique participants for each dataset, using 3-month bins.

**Appendix 1—figure 2.**
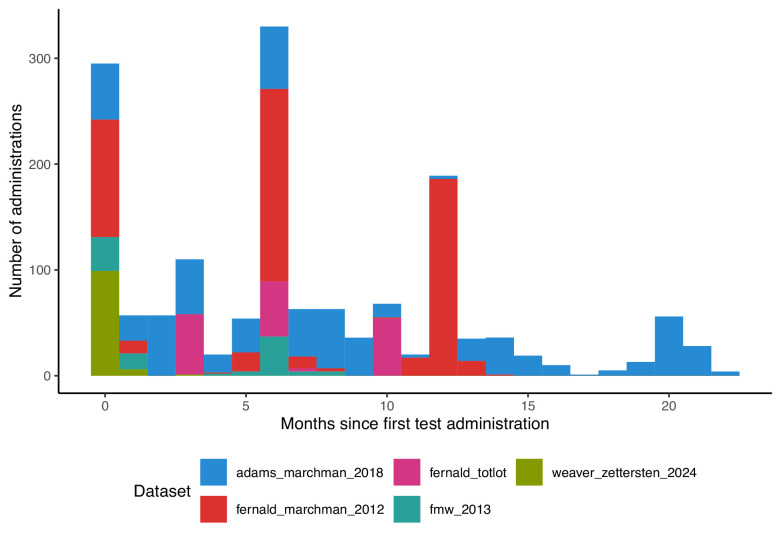
Distribution of retest administrations across datasets with repeated measurements, colored by dataset. Each count indicates a retest administration (initial administrations are excluded). Administrations listed with a retest interval of 0 indicate retests within a month of the initial administration.

**Appendix 2—figure 1.**
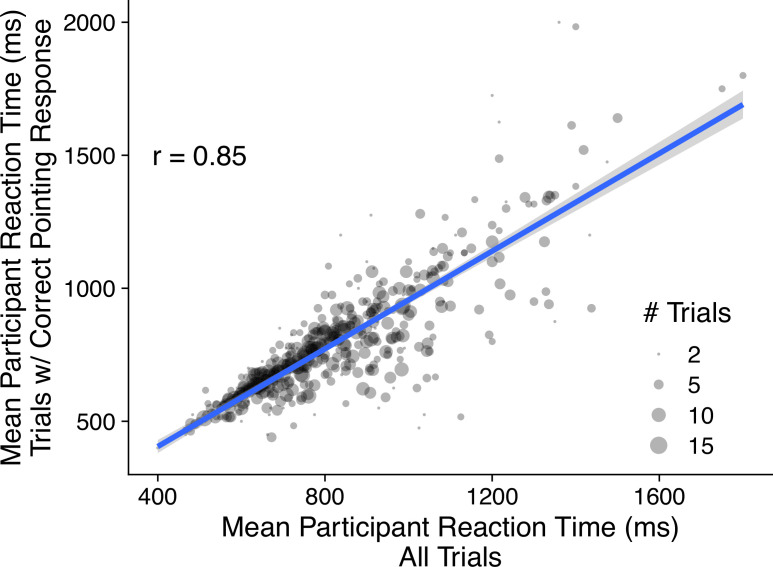
Correlation between reaction times on all trials and reaction times on trials where the child pointed to the correct target. Data from Creel, 2024.

**Appendix 3—figure 1.**
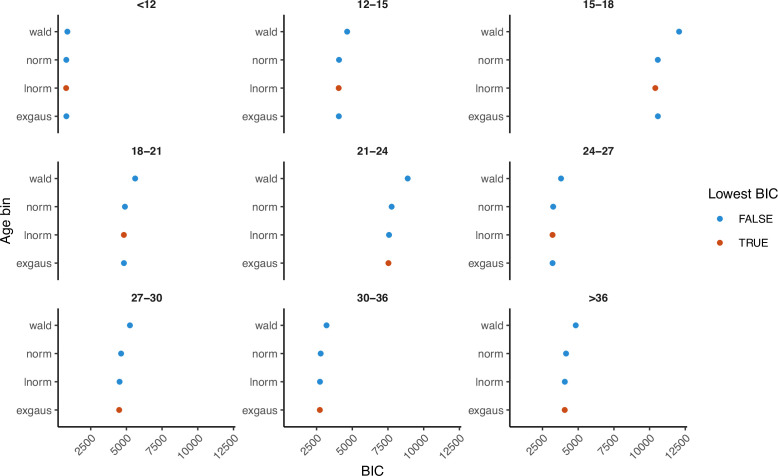
Goodness of fit for different distributional models for RT, split by age.

**Appendix 3—figure 2.**
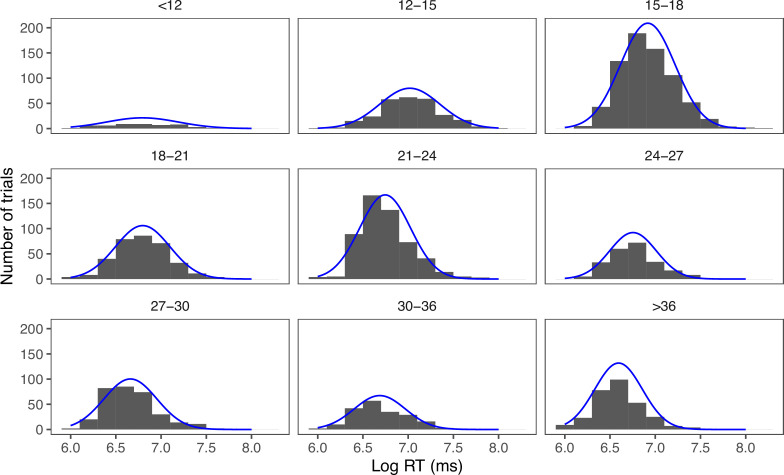
Distribution of RT overlaid with a log-normal distribution, split by age.

**Appendix 3—figure 3.**
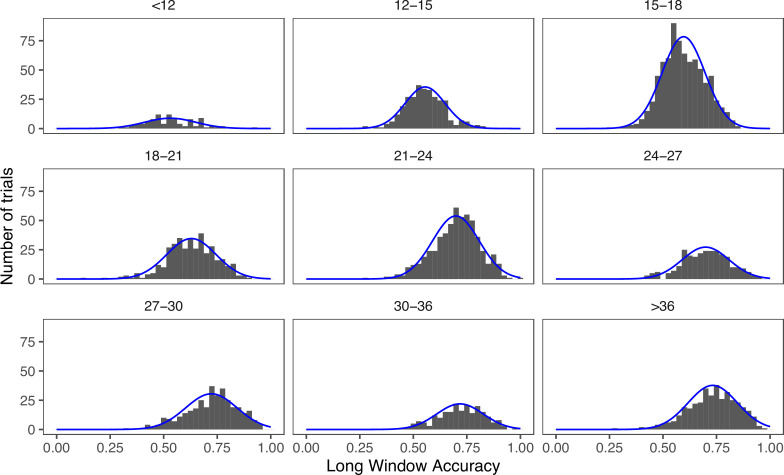
Goodness of fit for different distributional models of accuracy, split by age.

**Appendix 3—figure 4.**
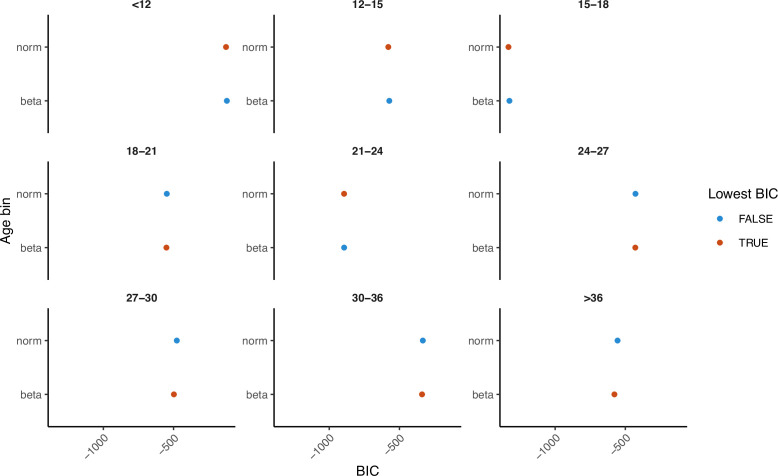
Distribution of accuracies overlaid with normal distribution, split by age.

**Appendix 9—figure 1.**
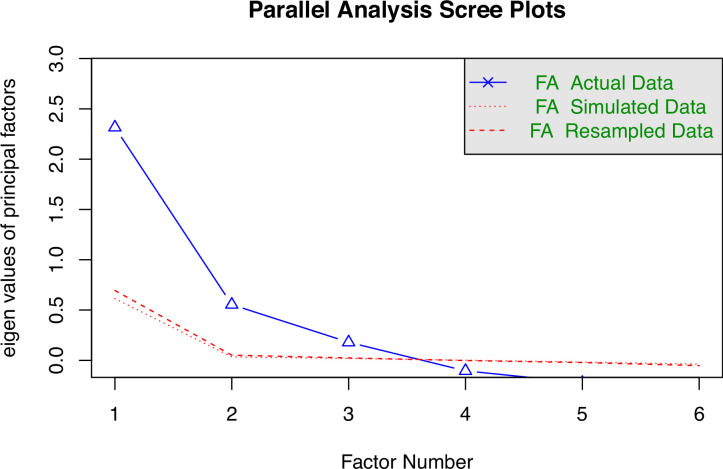
Parallel analysis scree plot showing the eigenvalues for each factor, for actual, simulated, and resampled data.

**Appendix 12—figure 1.**
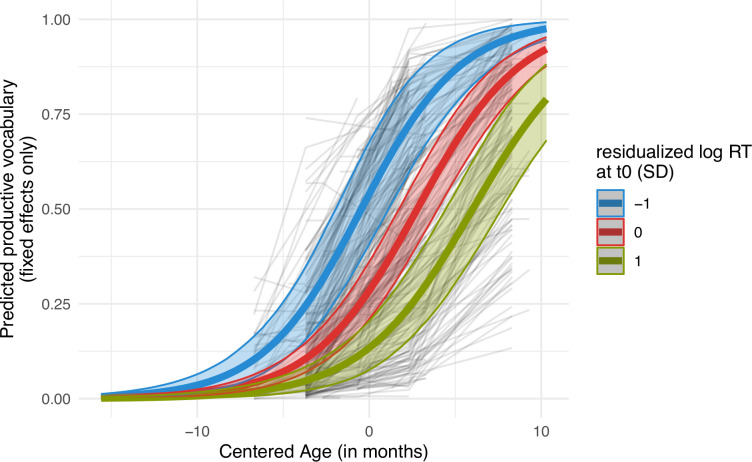
Growth curves from a logistic growth model showing predicted productive vocabulary growth for children based on their age-residualized initial reaction times. Predictions are shown for children with initial reaction times one SD faster than the mean for their age (blue), at the mean for their age (red), and one SD slower than the mean for their age (green). Individual longitudinal trajectories are shown in light gray. Solid lines show global model estimates and colored regions indicate 95% credible intervals.

## Data Availability

We retrieved all data from Peekbank release 2026.1 using the peekbankr R package. All code and data necessary to reproduce this manuscript are available at https://github.com/peekbank/peekbank-development (copy archived at Frank et al., 2026).

## Additional files

MDAR checklist

## Appendix 1

### Dataset description

Appendix 1—figure 1 gives the age distribution of unique participants for each separate dataset at different ages. Note that for some datasets, there are multiple administrations (i.e., experimental test sessions) for each participant. Appendix 1—figure 2 shows the distribution of measurement intervals for longitudinal studies within the dataset. Appendix 1—table 1 has additional information on how many trials each dataset contributed and what percent of the dataset’s trials were included.

**Appendix 1—table 1.** Characteristics of included datasets from Peekbank, sorted by what percent of the data they represent. Percent trials refers to what percent of the trials used came from that dataset; total is the number of trials used from that dataset; and included is what percent of all trials had data that was included (based on criteria about missingness, distractor to target transition, minimum RT). LW = long-window accuracy, SW = short-window accuracy, and RT = reaction time.

	Dataset name	Pct trials (LW)	Pct trials (RT)	Total trials (LW)	Total trials (RT)	Included (LW)	Included (SW)	Included (RT)
1	Adams et al., 2018	24.1%	27.1%	13146	5470	86.2%	89.3%	35.9%
2	Fernald and Marchman, 2012	20.1%	22.4%	10954	4526	91.6%	94.8%	38.0%
3	Weaver et al., 2024	8.0%	7.6%	4371	1540	88.6%	91.2%	31.3%
4	Fernald et al., 2013	7.4%	7.9%	4055	1603	83.1%	85.7%	32.9%
5	Fernald et al., 2006	6.4%	6.2%	3484	1254	93.1%	93.4%	34.0%
6	Bergelson and Swingley, 2012	2.9%	2.7%	1593	539	76.7%	83.5%	26.1%
7	Yurovsky et al., 2013	2.8%	2.2%	1544	450	53.2%	55.7%	17.2%
8	Borovsky and Peters, 2019	2.8%	0.0%	1520	0	88.9%	88.9%	0.0%
9	Potter and Lew-Williams, 2024	2.7%	2.6%	1453	523	89.3%	87.7%	32.1%
10	Yurovsky et al., 2017a	2.6%	2.4%	1411	493	60.4%	68.2%	21.9%
11	Yurovsky and Frank, 2017b	2.6%	1.9%	1401	393	65.3%	66.6%	20.9%
12	Weaver and Saffran, 2026	2.4%	2.4%	1309	475	67.5%	69.2%	24.5%
13	Yoon et al., 2015	2.0%	1.7%	1089	337	74.4%	77.1%	24.9%
14	Mahr et al., 2015	2.0%	2.0%	1073	395	82.4%	82.4%	30.3%
15	Garrison et al., 2020	1.7%	1.6%	944	320	89.8%	89.9%	30.4%
16	Ronfard et al., 2022	1.3%	1.4%	724	282	98.5%	98.5%	38.5%
17	Bacon and Saffran, 2022	1.3%	1.5%	687	304	90.4%	89.2%	40.0%
18	Perry and Saffran, 2017	1.2%	1.1%	649	228	89.6%	89.6%	31.5%
19	Pomper, R. & Saffran, J. R. (2017). Do infants learn to associate diminutive forms with animates? [unpublished raw data]. University of Wisconsin-Madison.	1.1%	0.9%	592	179	75.0%	75.8%	24.5%
20	Swingley and Aslin, 2002	1.1%	0.8%	585	159	98.2%	97.8%	28.0%
21	Frank et al., 2016	0.8%	0.7%	449	140	59.5%	66.2%	20.3%
22	Pomper and Saffran, 2016	0.8%	0.9%	457	175	95.6%	95.6%	37.9%
23	Moore and Bergelson, 2022	0.7%	0.7%	376	132	97.9%	99.2%	34.9%
24	Pomper, R. & Saffran, JR. (2015). Modulating attention to different features of objects during word learning [Unpublished Raw Data]*.* University of Wisconsin-Madison.	0.6%	0.6%	335	124	85.7%	89.0%	31.9%
25	Pomper and Saffran, 2019	0.4%	0.4%	213	75	85.9%	89.3%	30.5%
26	Pomper and Saffran, 2018	0.4%	0.3%	197	67	90.5%	91.9%	34.4%

## Appendix 2

### Reaction times

#### Appendix 2.1

##### Reaction time computation

Eye-tracking data are stored in Peekbank as a time series of fixations to specific areas of interest (in particular, the target and distractor on each trial). Other fixations can be to areas not in the target or distractor as well as to off-screen areas. This time series has a uniform sample rate of 25 ms/sample, based on resampling of the data in Peekbank to 40 Hz during preprocessing (Zettersten et al., 2023). Reaction times are computed by filtering trials to only those on which the child is fixating the distractor at the point of disambiguation ($t=0$) and then finding those trials on which the first non-missing fixation is to the target (hence excluding trials without a shift and trials on which a shift is to an off-screen location). The reaction time is then the total time from $t=0$ to the first timestep during which the child fixates the target. Consistent with standard practice in the literature following Fernald et al., 2008, RTs that are shorter than 367 ms are excluded as they are too short to be considered a response to the stimulus.

#### Appendix 2.2

##### Comparison of reaction times for correct and incorrect trials: Re-analysis of Creel, 2024

The Peekbank dataset only includes measurements of infants’ looking behavior, with no measure of a final target selection. This contrasts with work in the visual-world paradigm with older children and adults, in which participants make a final explicit choice about which image matches the target label (e.g., Colby and McMurray, 2023). Having this additional response allows a clearer separation of accuracy and reaction times because researchers can compute reaction times specifically on those trials in which participants responded correctly. This strategy helps avoid a possible mixing of reaction times for incorrect and correct responses, which might be generated by different underlying cognitive processes. A possible concern with the Peekbank datasets – and reaction times in infant looking-while-listening studies more generally – is that it is difficult to separate reaction times for correct versus incorrect responses in the absence of an independent final choice response.

To address this concern, we investigated data from a recent large-scale word recognition study with toddlers in which eyetracking measures were collected together with a final pointing response (Creel, 2024). This dataset included 914 responses from children (2.5–6.5 years) completing a looking-while-listening procedure in which they also were instructed to point to the target image. Using this dataset, we investigated the correlation between reaction times (following the same procedure as in our main analyses, i.e., focusing specifically on distractor-to-target shifts) computed over all trials and reaction times computed only for those trials in which children selected the correct referent. The results are shown in Appendix 2—figure 1. Reaction times (i.e., distractor to target shifts) for correct trials only were highly correlated with reaction times across all trials ($r=.85$, 95% CI [0.82, 0.87], $t(479)=34.84$, *p* < 0.001). This result suggests that having the ability to filter out incorrect trials has a minimal impact on reaction time computation, even in young children. While there is some uncertainty about how these results may generalize to infants in our younger age ranges (i.e., below 2.5 years of age), who struggle to provide reliable pointing responses, it seems reasonable to assume that our reaction time results would stay largely the same if it were possible to filter out trials on which infants make an incorrect mapping between the target label and the target image using an eyetracking-independent final choice response.

## Appendix 3

### Checks on data distributional assumptions

Here, we check whether the distributional forms that are assumed for the distributions of RT and accuracy are a reasonable empirical fit to the data, and compare against other commonly used distributional forms. We confirm that, across the age range, the choice to use a log-normal distribution for RT and a normal distribution for accuracy is justified.

### Appendix 3.1

#### Reaction time

The literature focuses on the use of the Exponential-Gaussian (ex-Gaussian) distribution as well as Wald, Weibull, gamma, and log-normal distributions (see for example Luce, 1986; Ratcliff, 1993; Van Zandt, 2002). All of these are two- or three-parameter distributions meaning that there is no necessary relationship between mean and variance.

The problem of fitting RT distributions is complex and a substantial literature exists (e.g., Ratcliff, 1979; Luce, 1986; Van Zandt, 2000; Harald Baayen and Milin, 2010). One of the big challenges in our dataset as well as elsewhere is that distributions are conditional on factors such as participant and task, so it is challenging to draw inferences about the underlying distribution when looking at average data.

That said, we find that overall the data are best fit by either an ex-Gaussian or a log-normal distribution, again consistent with prior literature, giving us confidence in this conclusion. Across the full dataset, the BIC values for ex-Gaussian (46,906) and log-normal (46,953) are quite close to one another, and better than the Wald (53,949) and normal (47,811) fits. When binned by age (Appendix 3—figure 1), younger children seem better fit for a log-normal distribution and older children seem better fit by ex-Gaussian (models with lowest BICs are shown in red since significant differences can be obscured by the large scale). Appendix 3—figure 2 shows the RT data distribution overlaid with the corresponding log-normal distributions. Overall, we think this result generally supports our decision to use log-transformed RTs as our primary dependent measure.

### Appendix 3.2

#### Accuracy

Individual trial-level accuracies are not binomial because they are an average probability of fixation over a viewing window. They are bounded at 0 and 1, but in general they tend toward the range 0.5–0.8 in most studies of this population. Appendix 3—figure 3 shows the data binned by age group with fitted Gaussian distributions.

These distributions seem well fit by standard Gaussians, but they are in principle bounded and so we asked whether this made a difference, using a Beta distribution (a two-parameter continuous distribution bounded at 0 and 1) for fitting. Surprisingly, across all data, the BIC values for the two distributions were very similar (–4710 for normal versus –4678 for Beta), though the normal distribution was slightly favored. Across age groups, there was heterogeneity with some groups better fit by a Gaussian and others better fit by a Beta (Appendix 3—figure 4). Again, we feel that this result generally supports our approach of modeling accuracies via standard linear mixed-effects models: their distributional form is quite close to normal.

## Appendix 4

### Test–retest reliability

We examined test–retest reliability for our primary variables of interest by calculating Pearson correlations between pairs of administrations given no more than 3 months apart. Test–retest correlations were significant but relatively modest: $\rho_{longwindowacc}=0.462$, $\rho_{shortwindowacc}=0.496$, $\rho_{rt}=0.407$. These reliabilities were biased downwards by three factors, however. First, longitudinal assessments sometimes use variable items between testing sessions, leading to item-related variance in measurement. Second, even 3 months can lead to substantial change in some children’s language abilities, thus correlations are attenuated by true change as well as measurement error. Third, longitudinal data in the dataset come primarily from the youngest children and hence are likely to show overall higher measurement error due to variability in children’s behavior and an overall lower number of trials.

## Appendix 5

### Pairwise correlations of main measures

Appendix 5—table 1 shows pairwise correlations between the primary variables of interest in the dataset.

**Appendix 5—table 1.** Pairwise correlations between primary variables of interest.

	age	log age	rt	log rt	long acc	short acc	prod	comp
age	1.00							
log age	0.98	1.00						
rt	–0.33	–0.35	1.00					
log rt	–0.34	–0.36	0.96	1.00				
long window accuracy	0.44	0.48	–0.48	–0.46	1.00			
short window accuracy	0.38	0.43	–0.62	–0.61	0.82	1.00		
production vocabulary	0.72	0.70	–0.31	–0.33	0.51	0.45	1.00	
comprehension vocabulary	0.42	0.42	–0.25	–0.24	0.24	0.24	0.59	1.00

## Appendix 6

### Functional form model comparison

Appendix 6—table 1 shows model comparison measures for different models of the functional form of the relationship between accuracy and age and Appendix 6—table 2 shows the same for reaction time. Age gradients are estimated substantially better with long window accuracies. Note that there are a greater number of observations for short window accuracies due to less missing data. We speculate that, on average, more participants looked away from the screen toward the end of trials, leading to a greater number of exclusions of long window trials based on the 50% criterion. Note that the total percentage of trials excluded is still small for both measures: 4.8% for long window accuracy and 1.8% for short window accuracy.

**Appendix 6—table 1.** Model comparison metrics for different functional forms of the relationship between accuracy and age.

Model	*n*. obs	sigma	logLik	AIC	BIC	REMLcrit	df.residual	*r* ^2^
Long window, linear age	55,337	0.272	–7012	14,038	14,100	14,024	55,330	0.124
Long window, log age	55,337	0.271	–6972	13,957	14,020	13,943	55,330	0.108
Short window, linear age	57,045	0.309	–14534	29,082	29,145	29,068	57,038	0.092
Short window, log age	57,045	0.309	–14,501	29,016	29,079	29,002	57,038	0.077

**Appendix 6—table 2.** Model comparison metrics for different functional forms of the relationship between RT and age.

Model	*n*. obs	sigma	logLik	AIC	BIC	REMLcrit	df.residual	*r* ^2^
Log RT, linear age	20,689	0.458	–13,844	27,703	27,758	27,689	20,682	0.232
Log RT, log age	20,689	0.457	–13,819	27,651	27,707	27,637	20,682	0.212
Linear RT, linear age	20,689	578.837	–161,573	323,159	323,215	323,145	20,682	0.197
Linear RT, log age	20,689	578.338	–161,546	323,106	323,161	323,092	20,682	0.185

## Appendix 7

### Power law fits

In the literature on the ‘law of practice’, although the log–log relationship we observed is commonly present in the aggregate across individuals, the situation is substantially more complex when relationships are measured within individuals. The best fitting curves for individuals are often exponentials or delayed exponentials (Evans et al., 2018; Heathcote et al., 2000).

With our current dataset, we unfortunately cannot specifically determine whether within-individual patterns of change conform to linear, power law, or exponential developmental patterns, because we have insufficient data about individuals’ improvement across time. Thus, our current results apply to the form of the age gradient as opposed to the form of any individual’s pattern of developmental change.

We believe that, unlike the skills being studied in the prior adult literature (e.g., Anderson, 1982; Heathcote et al., 2000; Logan, 1988), language processing is being learned over the course of a child’s lifetime. Thus, we do not expect to see within-paradigm changes in learning in what is a narrow period of time compared to the duration over which language processing skills are refined.

Nevertheless, here we test for other forms of the aggregate relationship between age and reaction time. In particular, we consider (1) a log–log relationship between RT and age (presented in the main text), (2) using both a log age and a linear age to predict log RT, (3) a quadratic relationship between age and RT, and (4) a cubic relationship between age and RT. As shown in Appendix 7—table 1, the model with a linear age term in addition to a log age term has the best fit, although the linear age term coefficient is not significant (coefficients in Appendix 7—table 2).

These models reveal a small and non-significant additional linear age term over and above log age, but – because individual participant-level fits are not possible – this term cannot really be used to weigh in on the debate about the precise nature of the learning pattern.

**Appendix 7—table 1.** Goodness of fit comparison between different models of the relationship between age and RT.

Model	*n*. obs	sigma	logLik	AIC	BIC	REMLcrit	df.residual	*r* ^2^
Log RT, log age	18,940	0.451	–12,393	24,801	24,855	24,787	18,933	0.205
Log RT, log + linear age	18,940	0.449	–12,349	24,719	24,806	24,697	18,929	0.216
Linear RT, poly(age,2)	18,940	569.500	–147,594	295,204	295,267	295,188	18,932	0.180
Linear RT, poly(age,3)	18,940	569.005	–147,567	295,151	295,222	295,133	18,931	0.188

**Appendix 7—table 2.** Fixed effects coefficients for a model predicting log RT from both log age and linear age.

Term	Estimate	Std. error	df	*t* value	p-value
Intercept	6.829	0.026	20.104	266.137	0.000
log age	–0.251	0.072	12.851	–3.515	0.004
linear age	0.132	0.078	13.232	1.697	0.113

## Appendix 8

### Mixed-effects model specifications

Here, we provide specifications for the lmer mixed-effects models used in the main text. These models are used to estimate the relationship between age and the primary variables of interest, controlling for dataset and subject-level variability.

For accuracy, four models were run, crossing long and short windows as the dependent variable with age or log age as the predictor.

long_window_accuracy ~age_s + (age_s | dataset_name) + (1 | subject_id)
long_window_accuracy ~log_age_s + (log_age_s | dataset_name) + (1 | subject_id)
short_window_accuracy ~age_s + (age_s | dataset_name) + (1 | subject_id)
short_window_accuracy ~log_age_s + (log_age_s | dataset_name) + (1 | subject_id)

For reaction time, four models were run, crossing rt and log rt as the dependent variable with age or log age as the predictor.

log_rt ~age_s + (age_s | dataset_name) + (1 | subject_id)
log_rt ~log_age_s + (log_age_s | dataset_name) + (1 | subject_id)
rt ~age_s + (age_s | dataset_name) + (1 | subject_id)
rt ~log_age_s + (log_age_s | dataset_name) + (1 | subject_id)

To look at the relationship between variance in the accuracy and reaction time measures and children’s age, we ran two models.

long_window_acc_var ~log_age_s + (log_age_s | dataset_name) + (1 | subject_id)
log_rt_var ~log_age_s + (log_age_s | dataset_name) + (1 | subject_id)

In the growth curve analysis, we fit a mixed-effects model predicting growth in vocabulary as a quadratic function of age, RT at study initiation (t0), and their interaction, using the formula below.

prod ~poly(age_15,2) *rt_t0 + (age | subject_id) + (1 | dataset_name)

## Appendix 9

### Factor analysis

Figure 3 shows the result of a parallel analysis supporting the presence of three factors in the exploratory factor analysis. Appendix 9—table 1 shows the factor loadings for the exploratory three-factor solution using varimax rotation. The first factor is primarily driven by vocabulary measures, the second by reaction time, and the third by accuracy measures.

**Appendix 9—table 1.** Factor loadings for the exploratory three factor solution using varimax rotation.

	F1	F2	F3
RT	–0.19	0.81	–0.30
RT var	–0.10	0.81	–0.22
long window accuracy	0.33	–0.31	0.55
long window accuracy var	–0.10	0.26	–0.65
production vocabulary	0.95	–0.04	0.30
comprehension vocabulary	0.61	–0.16	–0.01
age	0.63	–0.14	0.37

The confirmatory factor analysis of this three-factor solution was fit using the following specification:

vocab = ~prod + comp
accuracy = ~long_window_accuracy +long_window_acc_var
speed = ~log_rt +log_rt_var

The confirmatory factor analysis of the three-factor solution with a relation to age was fit using the following specification:

vocab =~prod + comp
accuracy =~acc + acc_sd
speed =~log_rt +log_rt_sd
vocab ~log_age
accuracy ~log_age
speed ~log_age

The SEM with a linear growth curve used the following specification:

accuracy_intercept =~1*acc_t0+1*acc_t1+1*acc_t2+1*acc_t3+1*acc_t4
accuracy_slope =~1*acc_t0+2*acc_t1+3*acc_t2+4*acc_t3+5*acc_t4
speed_intercept =~1*log_rt_t0+1*log_rt_t1+1*log_rt_t2+1*log_rt_t3+1*log_rt_t4
speed_slope =~1*log_rt_t0+2*log_rt_t1+3*log_rt_t2+4*log_rt_t3+5*log_rt_t4
vocab_intercept =~1*prod_t0+1*prod_t1+1*prod_t2+1*prod_t3+1*prod_t4
vocab_slope =~1*prod_t0+2*prod_t1+3*prod_t2+4*prod_t3+5*prod_t4
accuracy_intercept ~~NA*accuracy_intercept
accuracy_slope ~~NA*accuracy_slope
speed_intercept ~~NA*speed_intercept
speed_slope ~~NA*speed_slope
vocab_intercept ~~NA*vocab_intercept
vocab_slope ~~NA*vocab_slope

## Appendix 10

### Factor analysis on first administrations

As a robustness check, we tested our best factor analytic models using only cross-sectional data (filtering to the first test session in longitudinal datasets; *N* = 1963 instead of *N* = 3553). A comparison of all 4 models is shown in Appendix 10—table 1. For the three-factor CFA, the first administration model shows increased CFI (0.992 instead of 0.980) and decreased RMSEA (0.033 instead of 0.058). The same is true for the age-regressed three-factor CFA, which shows very good statistics on both first administrations and longitudinal data (CFI = 0.997 and 0.993, respectively, and RMSEA = 0.021 and 0.033, respectively).

**Appendix 10—table 1.** Comparison of confirmatory factor analysis models on longitudinal data or first administrations only.

Model	CFI	RMSEA	RMSEA_Lower_	RMSEA_Upper_	TLI	SRMR	AIC	BIC
No age, longitudinal	0.980	0.058	0.047	0.069	0.949	0.033	50,821	50,952
No age, first admin	0.992	0.033	0.018	0.049	0.980	0.023	27,464	27,585
Age, longitudinal	0.993	0.033	0.024	0.042	0.984	0.021	48,565	48,717
Age, first admin	0.997	0.021	0.007	0.034	0.993	0.013	26,173	26,313

## Appendix 11

### Alternative factor structures

In this section, we provide comparisons between the three-factor model we report in the main text and several alternative models, including:

- a one-factor model;
- a two-factor model with vocabulary separated from speed and accuracy;
- a two-factor model with speed separated from accuracy and vocabulary; and
- a two-factor model with variability terms separated from speed, accuracy, and vocabulary.

Appendix 11—table 1 shows the result of these comparisons. The three-factor model shows the lowest AIC and BIC, as well as being significantly better fitting than the next-best model.

**Appendix 11—table 1.** Model comparison for alternative factor structures. *p*-values show differences between adjacent models; no *p*-values are shown for comparisons between non-nested models.

	df	AIC	BIC	Chisq	Chisq diff	RMSEA	df diff	Pr(>Chisq)
Three-factor	6	46,346.69	46,475.43	91.96				
Two-factor (vocab)	8	46,397.74	46,514.22	147.01	55.05	0.09	2	<0.0001
Two-factor (speed)	8	46,486.96	46,603.44	236.23	89.22	0.00	0	
Two-factor (variability)	8	46,536.10	46,652.58	285.37	49.14	0	0	
One-factor	9	46,535.49	46,645.84	286.76	1.39	0.01	1	0.24

## Appendix 12

### Non-linear growth models

**Appendix 12—table 1.** Fixed effects estimates from logistic growth model.

Type	Estimate	Est. error	l-95% CI	u-95% CI
Constant component of growth intercept	2.850	0.606	1.680	4.109
Effect of*t*_0_ RT on growth intercept	3.187	0.604	1.970	4.369
Constant component of growth scale	1.121	0.058	1.009	1.242
Effect of*t*_0_ RT on growth scale	–0.026	0.079	–0.178	0.133

**Appendix 12—table 2.** Fixed effects estimates from logistic growth model using RT residualized on age as the predictor.

Type	Estimate	Est. error	l-95% CI	u-95% CI
Constant component of growth intercept	2.798	0.570	1.740	3.987
Effect of residualized*t*_0_ RT on growth intercept	3.237	0.562	2.119	4.340
Constant component of growth scale	1.104	0.061	0.986	1.232
Effect of residualized*t*_0_ RT on growth scale	0.056	0.079	–0.097	0.214

To test for the differentiation of vocabulary growth based on initial reaction time, we used the package brms to fit a (Bayesian) logistic growth model to the production data. This model has two parameters for the logistic curve, a scale and an intercept. Both were allowed to interact with initial reaction time. We also included random effects of logistic intercept and scale by participant and a grouping term across datasets. Age and initial reaction time were both mean-centered. This model showed a significant effect of initial reaction time on the intercept of the logistic growth curve, but not on its scale (see Appendix 12—table 1).

The formula specification was

nlform <- brms::bf (
prod ~1 / (1+exp((xmid - age_c) / exp(logscale))),
xmid ~1 + log_rt_0_c + (1 | dataset_name/subject_id),
logscale ~1 + log_rt_0_c + (1 | dataset_name/subject_id),
# scale ~1 + log_rt_0_c,
nl = TRUE
)

And the priors were

priors <- c (
prior(normal(0, 5), nlpar = "xmid", coef = "Intercept"),
prior(normal(1, 1), nlpar = "logscale", coef = "Intercept"),
prior(normal(0, 1), nlpar = "logscale", coef = "log_rt_0_c"),
prior(exponential(1), class = "sigma"),
prior(normal(0, 2), nlpar = "xmid", coef = "log_rt_0_c"),
# Random effects for xmid
prior(exponential(1), class = "sd", nlpar = "xmid", group = "dataset_name"),
prior(exponential(1), class = "sd", nlpar = "xmid", group = "dataset_name:subject_id"),
# Random effects for scale
prior(exponential(1), class = "sd", nlpar = "logscale", group = "dataset_name"),
prior(exponential(1), class = "sd", nlpar = "logscale", group = "dataset_name:subject_id")
)

Age and reaction time are correlated, so to check that the effects of initial reaction time were not due to age effects, we reran the model using residualized reaction time to remove effects of age. As seen in Appendix 12—table 2 and Appendix 12—figure 1, the pattern of effects is similar for residualized reaction time as for reaction time.

Interpretation of growth in both this model and the linear growth model in the main text is complicated by the fact that the CDI form puts a ceiling on the total number of words that can be recorded; both the quadratic growth functions and the logistic functions come together at the form ceiling. Thus, a shift in quadratic growth in the linear model and a shift in intercept in the logistic model both point to the same overall effect, which is faster growth at the point of maximal sensitivity of the CDI. Neither model can estimate whether the overall growth trajectory is different beyond the range of the CDI. Thus, although these models might initially seem to be in conflict, we believe that they actually point to the same phenomenon, which is perhaps better described by the longitudinal SEM model reported in the main text. Children with greater skill in word recognition show an overall positive shift in the growth trajectory of vocabulary development.

## Appendix 13

### SEM longitudinal missingness

The SEM model was fit to the entire dataset, including the large mass of cross-sectional data (to anchor the estimates of t0 coefficients) and the sparse longitudinal data for each time point. We have 3–12% of the total t0 datapoints for any given time point (see Appendix 13—table 1), given the sparsity of longitudinal sampling (only 5/26 of the datasets are longitudinal).

Our data are MAR (missing at random) rather than MCAR (missing completely at random). This is because their missingness is due to which dataset they are part of – if they are from a cross-sectional dataset, they are by definition missing all longitudinal observations. For our analyses to be appropriate given this structure, we have to assume that the general developmental patterns we are studying are replicated across datasets. We believe that they are, and we show this statistically using our mixed-effects and non-linear mixed-effects models, which control for dataset-related variation. We also show dataset-level effects in a number of our visualizations for this same reason. The same degree of random effect specification that we can do in the mixed-effects models is not possible in the SEM model, however, purely for technical reasons. Again, this point highlights the importance of convergence across analyses.

**Appendix 13—table 1.** Fraction of data present for each measure at each time point for the longitudinal SEM.

Time point	Log RT	Accuracy	Production	Comprehension
*t*0	0.685	0.865	0.279	0.183
*t*1	0.035	0.039	0.024	0.013
*t*2	0.093	0.096	0.095	0.000
*t*3	0.028	0.029	0.027	0.000
*t*4	0.031	0.032	0.031	0.000
