# Supplementary material for: Continuous developmental changes in word recognition and language learning across early childhood
Source: eLife. 2026 Jul 7;14:RP109636. doi: 10.7554/eLife.109636 (PMC13341113; doi:10.7554/eLife.109636)
Supplement: Peer review history

# Editor report: eLife Assessment

**DOI:** 10.7554/eLife.109636.3.sa0

**Authors:** Clare Press

**Affiliations:** University College London United Kingdom

**Keywords:** Compelling, Important

This is an **important** contribution that confirms prior evidence that word recognition — a cornerstone of development — improves across early childhood and is related to vocabulary growth. This study is distinguished by its use of a large, multi-study dataset that is uncommon in prior research on cognitive development. It provides **compelling** evidence that speed, accuracy, and consistency of word learning improve with age, and will therefore prove of interest to those studying language, and more broadly, perception and development.

# Referee report: Reviewer #1 (Public review)

**DOI:** 10.7554/eLife.109636.3.sa1

Summary:

The study examined the extent to which children's word recognition skill improves across early development, becoming faster, more accurate and less variable, and the extent to which word recognition skill is related to children's concurrent and later vocabulary knowledge.

The main strength of the study comes from the dataset which recycles previously collected data from 24 studies to examine the development of word recognition skill using data from 1963 children. This maximizes the impact of previously collected data while also allowing the study to reliably ask big picture questions on the development of word recognition skill and its relation to chronological age and vocabulary knowledge. Data analysis is rigorous, thought through and very clearly described. Data and code necessary to reproduce the manuscript are shared on the project's Github. The limitations of the study are acknowledged and the manuscript does well to tone down the causal implications of their results.

# Referee report: Reviewer #2 (Public review)

**DOI:** 10.7554/eLife.109636.3.sa2

Summary:

This paper presents a series of analyses of a large dataset combining many prior studies of early word recognition (Peekbank). The analyses demonstrate that the speed, accuracy and consistency of word learning improves with age. Moreover, the speed of word learning early in development was related to vocabulary growth over time.

Strengths:

A key strength of the paper is the use of a large multi-study dataset. This is particularly valuable in the field of early cognitive development, which has (due to practical limitations) often been based on small-scale studies that necessarily provide a shaky foundation for conclusions. The analyses are also well-motivated.

Weaknesses:

In an earlier version of the manuscript, the meaning of "word recognition ability" was ambiguous and could have referred to either (A) an intrinsic ability that matures, or (B) knowledge of the common, concrete words typically used in these studies that increases with experience. The revised version of the manuscript identifies these two interpretations and acknowledges that they cannot be teased apart in the current work.

# Author comment: Author response

**DOI:** 10.7554/eLife.109636.3.sa3

**Authors:** Michael C Frank, Virginia A Marchman, Claire Augusta Bergey, Veronica Boyce, Mika Braginsky, George Kachergis, Jess Mankewitz, Stephan C Meylan, Benjamin Prystawski, Nilam Ram, Robert Z Sparks, Adrian Steffan, Alvin Wei Ming Tan, Martin Zettersten

**Affiliations:** Stanford University Stanford United States; Stanford University Stanford United States; Stanford University Stanford United States; Stanford University Stanford United States; Stanford University Stanford United States; Stanford University Stanford United States; University of Wisconsin, Madison Madison United States; University of California, Berkeley Berkeley United States; Stanford University Stanford United States; Stanford University Stanford United States; Stanford University Stanford United States; Ludwig Maximilian University Munich Germany; Stanford University Stanford United States; University of California, San Diego San Diego United States

The following is the authors’ response to the original reviews

**General note**

We have issued a new release of the general Peekbank database, 2026.1, which includes more data integrity checks and several more datasets. As a result of this release, the underlying dataset we use in our paper has shifted slightly. The shifts represent a relatively small proportion of the total data and thus these changes have caused only relatively minor changes to our numerical results. We also highlight that we now include a small amount of data regarding children younger than 12 months, increasing the developmental range of our analysis (see Figure 1).

**Reviewer 1 (Public review):**

The limitations of the study are acknowledged to some extent, but need to be improved and ensured that they run throughout the manuscript. Thus, in the discussion, the authors note that the approach is observational and exploratory, and highlight for me a key alternative explanation of the findings, namely that faster children could be faster due to their larger vocabulary, rather than faster children learning more words. Indeed, the latter explanation for the relationship is called into question, given that growth in speed was not related to growth in vocabulary. Here, the authors note that the null result may be related to the fact that they do not sufficiently precise estimates of growth slopes, rather than taking the alternative explanation seriously that there may not be as causal a link between being a faster word learner and a better word learner (learn more words).

Thank you very much for your challenging and thoughtful comments. In hindsight we did not realize that the way we were writing about our results was ambiguous between several interpretations (one of which we endorse and one of which we do not).

We respond below to the specific suggestions about causal directionality in the longitudinal analysis, but we certainly believe that we cannot draw strong conclusions about causality from our dataset and have attempted throughout the paper to remove causal language that might have crept into our interpretation.

In response to your comments, we have made a number of key revisions aimed at qualifying and clarifying our points:

- The abstract now prominently notes that our design is observational: “In an observational study…”
- The abstract notes a positive and a negative result in the relationship between word recognition and vocabulary: “Further, across a range of longitudinal models, speed, accuracy, and vocabulary were coupled. Children with overall faster word recognition tended to show faster vocabulary growth, though developmental growth in word recognition skill was not specifically associated with growth in vocabulary.”
- The abstract removes potential casual language in the final sentence: “... these findings support the view that word recognition is a skill that develops gradually across early childhood and that this skill is deeply intertwined with early language learning.”
- A new paragraph in the Results introduces the potential hypotheses investigated via the longitudinal models.
- The final paragraph of the Results section sharpens the contrast between two possible growth hypotheses: “However, we did not find evidence for the stronger version of this claim: in neither the non-linear growth model nor the linear SEM did we find evidence that increases in speed were related to increases in vocabulary size. Thus, our findings do not support a ‘virtuous cycle’ model in which increases in recognition specifically lead to increases in vocabulary size.”

We hope these changes lead to a manuscript that better aligns with the limitations of the study.

This is especially since, but correct me if I’m wrong here, the current vocabulary size is not taken into consideration in the model examining vocabulary growth. Given the increasing number of studies showing that current vocabulary knowledge predicts vocabulary growth (Laing, Kalinowski et al, Siew & Vitevitch), one simple alternative explanation is that current vocabulary knowledge predicts both current word recognition skill and later vocabulary knowledge. Is there anything in the data speaking against this hypothesis?

We think the reviewer’s overall point is generally correct, as we described above, but we want to clarify a specific statistical point. The non-linear longitudinal model of vocabulary growth does in fact take into account a child’s average vocabulary size. (This point feels tricky in a non-linear model but it’s actually quite similar to a linear model for the purposes of this discussion). Basically, vocabulary (at all timepoints) is modeled as a function of age, with both main effects and interactions with age. Critically, each participant is also modeled as having a random intercept capturing their deviation from the average growth pattern across ages (as expressed by the fixed effects). In this model, the “main effect” (here captured by the intercept for the logistic curve in the model) that we observe for speed indicates that vocabulary growth for individuals is predicted to be faster (their curve is shifted left) if their RTs are fast. The presence of the random effects in this model thus “controls” for the fact that some participants have overall higher vocabularies (and are shifted up relative to the average growth curve).

But, we note that this model does not show an “interaction effect” (here captured by the null effect of RT on the slope parameter in the logistic model). That’s one of the null effects that we now call out much more prominently in the abstract and end of the results (per our response above).

Equally, while the SEM examines vocabulary growth controlling for age, I wonder about the other way around. What would happen to the effect of age on word recognition skill (in the LME model, S8) if one were to add concurrent vocabulary size? So does chronological age explain word recognition skill or vocabulary knowledge? Right now, the manuscript describes this effect purely related to chronological age, but is it age per se or other cognitive abilities, including a key change across development, namely, vocabulary size? Thus, the presentation of the skill learning hypothesis suggests that age is a proxy for experience, while you actually have here a very nice proxy for experience in terms of children’s vocabulary size.

Again, thank you for engaging with this tricky set of issues. Overall, our goal is to adjust the manuscript to reflect points of agreement; in particular, we agree that age is a proxy for language experience, vocabulary, and other cognitive changes, and we have stated this explicitly now in the intro to the factor analyses: “In our prior analyses, chronological age acts as a proxy for greater language experience and larger vocabulary as well as a host of other correlated developmental changes in cognition. Now we explicitly explore relations to vocabulary growth and the triadic relationship between age, word recognition, and vocabulary.”

On the statistical side, we do think that the NLME (non-linear mixed effects; the logistic growth mode) effectively controls for average vocabulary size, as described above. The longitudinal SEM also relates vocabulary growth to growth in word recognition skill. In both models, we find no evidence for coupled growth; instead the evidence points to children with higher baseline word recognition skill showing faster growth in vocabulary (speed intercept significantly related to vocabulary slope, -.14, *p* < .01) but not the reverse (vocabulary intercept not strongly related to speed slope; -.01, *ns*).

More generally, we hope our edits to the paper, detailed above, both clarify this tricky set of issues and also remove inappropriate casual language throughout.

Critically, while the discussion is more nuanced, the way the abstract is concluded and the way the Introduction is phrased suggest that the study is able to answer a causal question, which, as the authors themselves note, is not possible. The abstract, for instance, states that word recognition becomes faster, more accurate and less variable...consistent with a process of skill learning. And also that this skill plays a role in supporting early language learning, which is very causal language. I don’t think you can really claim that you are testing the two hypotheses you suggest here. The work is definitely embedded in the context of these hypotheses, but are you really able to test them? My worry is that while the discussion is more nuanced, the extent to which this study will then be cited down the line as showing that children learn more words down the line because they are faster at recognizing words, and anything that you can do to tamper with such interpretations would be good for the literature. For me, this should not just be relegated to the discussion but should be touched upon in the abstract and Introduction.

Thanks for pushing us to be more precise with how we frame and describe our findings. We agree with the reviewer that our findings do not warrant strong conclusions about the causal role of word recognition skill in vocabulary growth. Per our response above, we have now tried to carefully revise our language throughout the paper (in particular, in the abstract and introduction, as noted by the reviewer).

Finally, it would help to talk more about the mechanisms at work in any relationship between word recognition and language learning. It seems to me that this would rely on some predictive processing framework, given the description on page 4, and it would be good to make this clear (faster and more accurately you can recognize a ball, better use this evidence to infer the speaker’s intended meaning).

Thanks, this is a great point. We’ve revised this text and added references to predictive processing, unpacking a problematic paragraph into two:

“Familiar word recognition -- as measured by LWL -- is hypothesized to play a key role in language learning (19). The idea, in a nutshell, is that the faster and more accurately a child can process incoming words, the more opportunities they have for learning. Consider a child hearing the utterance "Can you put the ball in the crate?" The better the child can recognize the word "ball", the better they can use this evidence to help infer the speaker's intended meaning, allowing possible inferences about the meaning of the less familiar word, "crate" (20).

“Real time language processing, including word recognition, relies heavily on predictive processing, in which comprehenders integrate expectations from prior linguistic context with noisy and ephemeral incoming signals (21, 22). The more input a child receives, the better their predictions are likely to be, and hence the more they can learn (19, 23). Indeed, measurements of children's language input at home are consistently associated with their vocabulary size (24, 25). And, in line with this predictive processing framework, one important study found that children's word recognition speed mediated the longitudinal relationship between home language input and vocabulary growth (26). Thus, word recognition is thought to be a key support for ongoing word learning.”

Equally, when referring to word recognition, it would be good to clarify what this refers to - how well a child knows what a word refers to (and in the context of LWL, what it does not refer to) or how quickly it directs attention to what is referred to.

Thanks, we’ve added a capsule definition in the second paragraph, and added the sentence “This procedure [LWL] measures the general construct of word recognition by operationalizing knowledge of a meaning as visual attention to a specific named referent.” We hope this clarifies the relationship between LWL and word recognition.

With regards to the data, I wonder if there is a clustering of kids past 24 months that is happening here, looking at Figures 1 and 2, where it seems like there is less change past the 24-month point. Is there any way to look at whether the effect of age or vocabulary on word recognition is not linear but asymptotic?

Thanks for pointing this out; we do see what you are talking about but think it’s being handled appropriately in the analysis. In Figure 1 it clearly looks like changes to RT are asymptotic – this is why we analyze the logarithm of RT throughout the paper. In Supplement S6 we show that reaction time is indeed best fit by a log-log function. Your question about Figure 2 asks whether there is further structure beyond the log-log fit; in Supplement S7 we show some analyses that suggest a polynomial fit is not better than the log-log fit; there is some small additional linear effect of age over and above the log-log fit, but it’s minor and pretty hard to interpret in our view.

**Recommendations for the authors:**

**Reviewer #1 (Recommendations for the authors):**

Page 3. Word production may manifest in overt behaviour but need not reflect complete knowledge. A child can say the word dog and use it to refer to a cat.

This is a good point. Since we are not able to speak to the precision of meaning representations (an important issue in its own right), we have omitted the phrase “with incomplete knowledge.”

Page 4. The first two sentences of the paragraph beginning with word recognition ability... don’t go together. The second sentence does not support the claim that word recognition plays a role in language learning.

Thanks, we’ve tried to smooth out this transition as part of unpacking the role of predictive processes.

Page 4. “predicts children’s standardized test scores years later” - make clear what test scores are here.

We added some additional details. The specific tests were the CELF (expressive language) and the KABC (IQ), but we thought too much detail might be distracting.

Page 5. I love Table 1, but would like for the data to be weighted somehow. So, given that some studies had a lot more trials and more children, what percentage of the data did this study contribute? That allows a clearer view of how biased the sample is in certain studies. The x in CDIS and longitudinal could be aligned to the right. I kept wondering why there was an x near some trials.

Thanks, we’ve adjusted the table to add the percentage of the total dataset (in trials) due to each study and fixed the alignment issue.

Page 6. 12 million individual samples: what samples are these? Individual data points per trial per time point. Making this clear would be great.

Clarified, thanks.

Page 9. Your accuracy measures only seem to consider the target. From what I remember of my preferential looking days, this measure usually also includes the distractor. Why do you not do this? This is especially since you have such a wide age range, so if a 12-month-old only looks for about 50 per cent of the trial and spends that time looking at the target, that is very different from a child who looks at the screen all of the trial and spends less time looking at the target here.

Sorry for any lack of clarity: we do in fact compute accuracy as the ratio of looking to target over looking to target plus looking to distractor. We have added this information to the parenthetical referenced above: “... accuracy (more target looking; computed as the ratio of target to target plus distractor looking)”.

Page 12. I only found out that age was in this model by looking at S9.

Thanks for mentioning this omission, we’ve clarified in the text: “We initially add age as an additional variable to our models to explore whether this factor structure relates to age; later we treat age as a predictor of latent factors.”

Page 12. Isn’t it trivial that speed and accuracy show negative covariance, especially given how you measure accuracy? Thus, if I take longer to fixate the target, I have less time to look at the target during the trial. If, however, I included the distractor in my accuracy measure, then I could still take longer to look at the target, but still look more at the target than the distractor.

Thanks for mentioning that this covariance is not the key result of interest; that observation didn’t come out in the text. Now we note that this covariation is “... as expected since they [speed and accuracy] are derived from the same data.” Note per above that accuracy is computed as target / target + distractor looking; even so, your observation is correct: slower looking at the target means lower accuracy at least to some degree.

Page 19. If you excluded data from trials with less than 50% of timepoints, how did this vary across age? Arguably, your study has to worry less about this, given your sample size, but it would be nice to know, which you could include in the percentage of data that each study contributed to the final sample.

Thanks, we’ve added this information to a new table in S1.

**Reviewer #2 (Public review):**

First, I wasn’t entirely clear about what the authors meant by “word recognition ability”. For much of the manuscript (including the use of the term “word recognition ability” itself), this comes across as an intrinsic ability or skill that improves with development. Alternatively, the speed and accuracy metrics taken from studies in Peekbank might capture children’s increasing knowledge of the common, concrete words typically used in these studies. To me, this is a somewhat different construct from a general skill at recognizing words. It would be helpful if the authors could clarify which construct they intend to capture, or if it is not possible to distinguish between these constructs from the Peekbank data.

In response to this comment and related comments above, we’ve added text to the first two paragraphs trying to clarify the general construct that we’re talking about – recognizing the meaning of a word in real-time language comprehension. We’ve also clarified several times throughout the introduction that we’re talking about familiar word recognition, that is, the ability to recognize specific known words. Further, we directly acknowledge the issue above in the introduction:

“Critically, most word recognition paradigms use words that children at the target age are reported to understand and produce. They are thus not indices of vocabulary size but rather measures of how quickly and accurately the child can recognize a familiar spoken word and use it to guide their visual attention to a referent. However, it is unknown the extent to which specific responses reflect an individual child's general speed of language processing versus their familiarity of specific words.”

Second, and relatedly, if the source of the age-related improvements is increasing experience with the common concrete words used in the Peekbank studies, then one might expect word recognition and improvements with age to be related to word frequency, given that more frequent words are experienced more often. Word frequency predicts word knowledge when assessed using CDI data. Can effects of frequency be detected in Peekbank word recognition metrics? If not, why? Similarly, is the speed and accuracy of word recognition in Peekbank data related to CDI-derived word age of acquisition, and again, if not, why?

This is a fascinating set of ideas, and one that we’ve pursued extensively using the Peekbank data. Unfortunately, we think it is out of scope for the current paper, which focuses on child-level metrics (including vocabulary and processing measures). Right now the current paper doesn’t include any analysis of individual words.

Just to expand a bit on the problem here: unfortunately, modeling word recognition as a simple linear function of (log) word frequency is only possible in the case that distractors are held constant (e.g., “ball” always has “book” as its distractor), because distractor frequency plays an important role in the recognition process. However, in our dataset, words are paired with many different distractors across studies. This property means a fairly complex model of the LWL decision process would be necessary for a model to successfully predict effects for individual words. While such a model is an exciting research goal, it’s not something we can include in the current manuscript.

Finally, there is a bit of a risk of the main findings of this paper coming across as a foregone conclusion. I.e., how could it be otherwise that word recognition improves with development?

**Reviewer #2 (Recommendations for the authors):**

Regarding the feedback about the risk of the findings coming across as a foregone conclusion - perhaps a primary place in the paper where it would be useful to clarify this point is on page 6, in the paragraph beginning, “We investigate two specific hypotheses here. First, one influential theory...”. Here, it might be worth clarifying whether there are alternative ideas about the emergence of word recognition in childhood that predict different patterns, so that the findings of the current paper can be framed as shedding new light on word recognition in development, rather than a confirmation of the common-sense idea that word recognition must improve over development.

Thanks, we appreciate this feedback and it’s something we’ve struggled with in this project. Our conclusion is that this paper does not constitute a binary hypothesis test of e.g., whether word recognition is linked to vocabulary development. Instead, we lean into the idea that there are empirical issues (rather than hypotheses) that have not been quantified sufficiently. Thus, we end the revised introduction with the following paragraph:

“Across both of these issues, the contribution of our work here lies in the detailed quantitative description of development. Nearly every theory of language learning assumes some role for continuous developmental change in word recognition, but these assumptions have not previously been anchored to specific measurements. Hence neither the functional form of the assumed changes nor their concurrent and predictive relationships to vocabulary have been quantified. We leverage the Peekbank dataset to accomplish these goals.”
